# Accumulation of p53 via down-regulation of UBE2D family genes is a critical pathway for cadmium-induced renal toxicity

**DOI:** 10.1038/srep21968

**Published:** 2016-02-25

**Authors:** Jin-Yong Lee, Maki Tokumoto, Yasuyuki Fujiwara, Tatsuya Hasegawa, Yoshiyuki Seko, Akinori Shimada, Masahiko Satoh

**Affiliations:** 1 Laboratory of Pharmaceutical Health Sciences, School of Pharmacy, Aichi Gakuin University, 1-100 Kusumoto-cho, Chikusa-ku, Nagoya, Aichi 464-8650, Japan; 2 Department of Environmental Health, School of Pharmacy, Tokyo University of Pharmacy and Life Sciences, 1432-1 Horinouchi, Hachioji, Tokyo 192-0392, Japan; 3 Department of Environmental Biochemistry, Mount Fuji Research Institute, 5597-1 Kenmarubi, Kamiyoshida, Fujiyoshida, Yamanashi 403-0005, Japan; 4 Laboratory of Pathology, Department of Medical Technology, School of Life and Environmental Science, Azabu University, 1-17-71 Fuchinobe, Chuo-ku, Sagamihara, Kanagawa 252-5201, Japan

## Abstract

Chronic cadmium (Cd) exposure can induce renal toxicity. In Cd renal toxicity, p53 is thought to be involved. Our previous studies showed that Cd down-regulated gene expression of the UBE2D (ubiquitin-conjugating enzyme E2D) family members. Here, we aimed to define the association between UBE2D family members and p53-dependent apoptosis in human proximal tubular cells (HK-2 cells) treated with Cd. Cd increased intracellular p53 protein levels and decreased *UBE2D2* and *UBE2D4* gene expression *via* inhibition of YY1 and FOXF1 transcription factor activities. Double knockdown of *UBE2D2* and *UBE2D4* caused an increase in p53 protein levels, and knockdown of *p53* attenuated not only Cd-induced apoptosis, but also Cd-induced apoptosis-related gene expression (*BAX* and *PUMA)*. Additionally, the mice exposed to Cd for 6 months resulted in increased levels of p53 and induction of apoptosis in proximal tubular cells. These findings suggest that down-regulation of UBE2D family genes followed by accumulation of p53 in proximal tubular cells is an important mechanism for Cd-induced renal toxicity.

Cadmium (Cd) is a chemical contaminant that induces serious tissue damage to the kidney and liver, and can result in cancer^1^,^2^,^3^,^4^. Orally-ingested Cd accumulates in many organs, particularly the kidney, and because of its long biological half-life (10–30 years)^3^,^5^, chronic Cd exposure at lower levels can influence kidney function. Cd is taken up by proximal tubular cells as a metallothionein-bound detoxified form^6^. This bound form is then metabolized in lysosomes and Cd is released^6^. Although unbound Cd stimulates metallothionein production in the kidney and then binds to metallothionein, the toxic effects occur when unbound Cd accumulates in proximal tubular cells^6^,^7^. At the molecular level, Cd has some effects on cellular proliferation, cell signaling, DNA repair and apoptosis^4^,^8^,^9^. However, the precise mechanism of Cd-induced proximal tubular cell toxicity remains unclear.

We have showed that Cd alters gene expression in several tissues *in vivo* and cultured cells, including proximal tubular cells^10^,^11^,^12^,^13^,^14^. In NRK-52E rat proximal tubular epithelial cells, Cd inhibited the gene expression of Ube2d4, a member of the UBE2D (ubiquitin-conjugating enzyme E2D) family^10^. UBE2D family is an E2 ubiquitin-conjugating enzyme family in the ubiquitin-proteasome system, through which proteins are modified with ubiquitin^15^. Our previous study demonstrated Cd decreased gene expression levels of not only Ube2d4 but also other UBE2D family members, Ube2d1, Ube2d2, and Ube2d3 in NRK-52E cells^16^. Ubiquitination is the post-translational modification of proteins and plays a critical role in the regulation of cellular processes including protein degradation, protein trafficking, DNA repair, and signal transduction^17^,^18^,^19^. The E2 ubiquitin-conjugating enzyme is the critical component in transferring the ubiquitin to target proteins in ubiquitin-proteasome system^20^. E2 ubiquitin-conjugating enzymes have been reported to be involved in cell viability when stressed by toxic materials^21^,^22^. Apollon protein containing ubiquitin-conjugating domain ubiquitinates apoptosis-related proteins in human embryonic kidney 293T cells, and Apollon-deficient MEFs (mouse embryonic fibroblasts) are more sensitive to apoptosis^23^. Moreover, E2 ubiquitin-conjugating enzyme is involved in amyloid-beta neurotoxicity through modulating ER-resident caspase-12^24^,^25^.

Several studies suggest the involvement of p53 in Cd toxicity in various cells^26^,^27^,^28^,^29^,^30^. The tumor suppressor p53 is involved in the inhibition of cell growth and apoptosis through transcriptional activity^31^. Cd is known to induce apoptotic cell death in various cell types^4^. Interestingly, UBE2D family is related to the ubiquitination of tumor suppressor protein p53 in human breast carcinoma MCF7 cells^32^. We demonstrated that Cd not only increased phospho-p53 level but also induced apoptosis in NRK-52E cells^16^. These observations suggest that Cd-induced apoptosis may be cause of the accumulation of p53 protein, which in turn may be cause of the down-regulation of UBE2D family genes. However, whether UBE2D family is involved in the degradation of p53, and whether p53 is actually associated with Cd-induced apoptosis in human proximal tubular cells remains to be elucidated.

In this study, we examined the effect of Cd on UBE2D family gene expression, the accumulation of p53 protein, and apoptosis in human proximal tubular cells (HK-2 cells). In addition, we monitored transcription factors involved in the Cd-regulated gene expression of the UBE2D family, the effect of the UBE2D family on p53 degradation, the effect of p53 on Cd-induced apoptosis, and the apoptotic effectors up-regulated by Cd-induced p53 stability using siRNA transfection. Finally, we exposed mice to 300 ppm Cd for 6 months to monitor p53 accumulation and apoptosis in proximal tubular cells of the mouse kidney.

## Results

### Cd induces p53 accumulation *via* suppression of *UBE2D2* and *UBE2D4* gene expression in HK-2 cells

**Figure 1:**
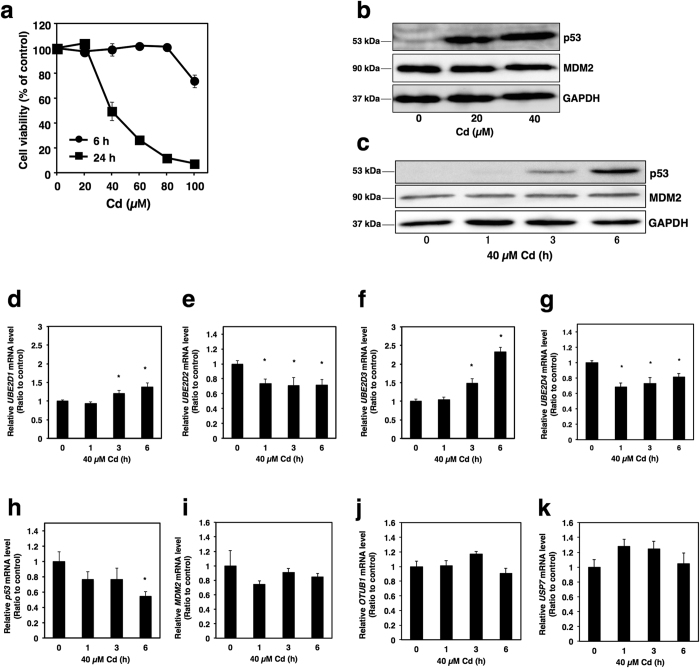
Effects of Cd on protein level of p53 and expression level of the genes related on p53 stability in HK-2 cells. (**a**) Cell viability of HK-2 cells after treatment with Cd for 6 or 24 h using the Alamar Blue assay. HK-2 cells were grown in 96-well plates at a density of 2.0 × 10^4^ cells/cm^2^ and cultured for 48 h. Culture medium was discarded and the cells were treated with Cd (CdCl_2_) in serum-free culture medium for 6 or 24 h. Values are the mean ± S.D. (*n* = 3). (**b**) Western blot analysis of p53 and MDM2 in HK-2 cells after treatment with Cd at 20 or 40 μM for 6 h. Anti-GAPDH antibody was used as a loading control. Upper panel, p53; middle panel, MDM2; lower panel, GAPDH. The three blots were run under the same experimental conditions. Uncropped images are provided in Supplementary Fig. 1a. (**c**) Western blot analysis of p53 and MDM2 in HK-2 cells after treatment with Cd at 40 μM for 0, 1, 3 and 6 h. Anti-GAPDH antibody was used as a loading control. Upper panel, p53; middle panel, MDM2; lower panel, GAPDH. The three blots were run under the same experimental conditions. Uncropped images are provided in Supplementary Fig. 1b. Real-time RT-PCR of *UBE2D1* (**d**), *UBE2D2* (**e**), *UBE2D3* (**f**), *UBE2D4* (**g**), *p53* (**h**), *MDM2* (**i**), *OTUB1* (**j**) and *USP7* (**k**) gene expression. HK-2 cells were grown in six-well plates at a density of 2.0 × 10^4^ cells/cm^2^ and cultured for 48 h. Culture medium was discarded and the cells were treated with Cd in serum-free culture medium for the indicated time. mRNA levels were normalized to *GAPDH*. Values are the mean ± S.D. (*n* = 3). *Significantly different from the corresponding control group, *P* < 0.05.

**Figure 2:**
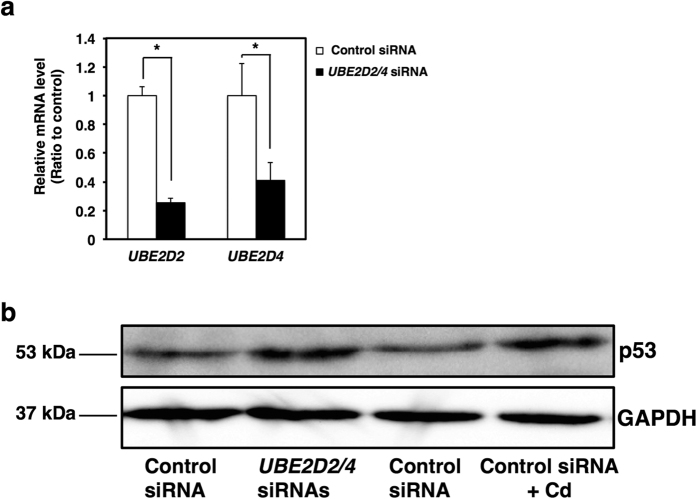
Cd increases p53 protein levels by suppressing *UBE2D2* and *UBE2D4* gene expression in HK-2 cells. (**a**) Knockdown efficiency of *UBE2D2* and *UBE2D4* genes in HK-2 cells by *UBE2D2* and *UBE2D4* siRNA treatment. *UBE2D2* and *UBE2D4* siRNAs were added to HK-2 cells for 24 h. mRNA levels were examined using real-time RT-PCR. mRNA levels were normalized to *GAPDH*. Values are the mean ± S.D. (*n* = 3). *Significantly different from the corresponding control group, *P* < 0.05. (**b**) p53 protein levels in *UBE2D2* and *UBE2D4* double knockdown HK-2 cells. Whole cell lysates of HK-2 cells treated with *UBE2D2* and *UBE2D4* siRNAs for 24 h or 40 μM Cd (CdCl_2_) for 3 h were used for western blotting and probed with p53 antibody. Anti-GAPDH antibody was used as the loading control. Upper panel, p53; lower panel, GAPDH. The two blots were run under the same experimental conditions. Uncropped images are provided in Supplementary Fig. 1c.

To investigate Cd-induced cytotoxicity in HK-2 cells, cell viability was determined in HK-2 cells using the Alamar Blue assay. HK-2 cells treated with 40 μM Cd for 24 h exhibited 50% cell viability; however, a 6 h treatment with 40 μM Cd did not induce cytotoxicity (Fig. 1a). Some reports suggest that Cd changes p53 protein levels and/or phosphorylated p53 protein levels in several cell types^33^,^34^,^35^,^36^,^37^. However, it remains unclear whether Cd increases intracellular p53 protein levels in HK-2 cells. Because 24 h treatment with Cd at 40 μM or greater causes severe cytotoxicity, the effect of Cd treatment for 6 h on cellular p53 protein levels was examined in HK-2 cells. Intracellular p53 protein levels were markedly increased following exposure to 20 and 40 μM Cd in HK-2 cells (Fig. 1b). Moreover, Cd-induced p53 accumulation was observed after a 3 h treatment (Fig. 1c). These results suggest that intracellular p53 protein accumulates in Cd-treated HK-2 cells before the appearance of cytotoxicity. As UBE2D family is involved in the stability of p53 in MCF7 cells^32^, we examined the effect of Cd on UBE2D family gene expression in HK-2 cells. Cd significantly decreased the expression of *UBE2D2* and *UBE2D4*, but not *UBE2D1* and *UBE2D3* in HK-2 cells (Fig. 1d–g). Because Cd did not increase *p53* mRNA levels (Fig. 1h), it is considered that post-transcriptional modification of p53 might be involved in the accumulation of p53 protein. Stability of the p53 protein is primarily regulated by the ubiquitin-proteasome system^33^,^34^,^38^. MDM2 is the principal E3 ubiquitin-ligase for the degradation of p53^33^,^34^,^38^. Previous studies have shown that the stability of p53 is also regulated by deubiquitinating enzymes such as Otub1 (OTU (ovarian tumor) deubiquitinase 1) and USP7 (ubiquitin specific peptidase 7)^36^,^37^. In the present study, Cd did not alter the cellular protein levels of MDM2 (Fig. 1b,c), nor mRNA levels of *MDM2* (Fig. 1i). Cd did not affect the mRNA levels of *OTUB1* and *USP7* (Fig. 1j,k), either. Therefore, Cd-induced p53 accumulation may be independent of MDM2 and the deubiquitinating system. A previous study also reported that p53 was degraded in cells from Mdm2 null mice^35^, suggesting that there are alternative regulators for the stability of p53. As shown in Fig 1e,g, gene expression of UBE2D2 and UBE2D4 was suppressed in HK-2 cells treated with Cd. To examine the direct involvement of *UBE2D2* and *UBE2D4* in the stability of p53, we next employed siRNA transfection against the genes *UBE2D2* and *UBE2D4* (Fig. 2a). Double knockdown of *UBE2D2* and *UBE2D4* increased intracellular p53 protein levels in HK-2 cells, as well as in cells treated with 40 μM Cd for 3 h (Fig. 2b). These observations indicate that Cd induces the accumulation of p53 protein mainly through the suppression of *UBE2D2* and *UBE2D4* transcription.

### Cd suppresses *UBE2D2* and *UBE2D4* transcription *via* inhibition of YY1 and FOXF1 transcription factor activity

**Figure 3:**
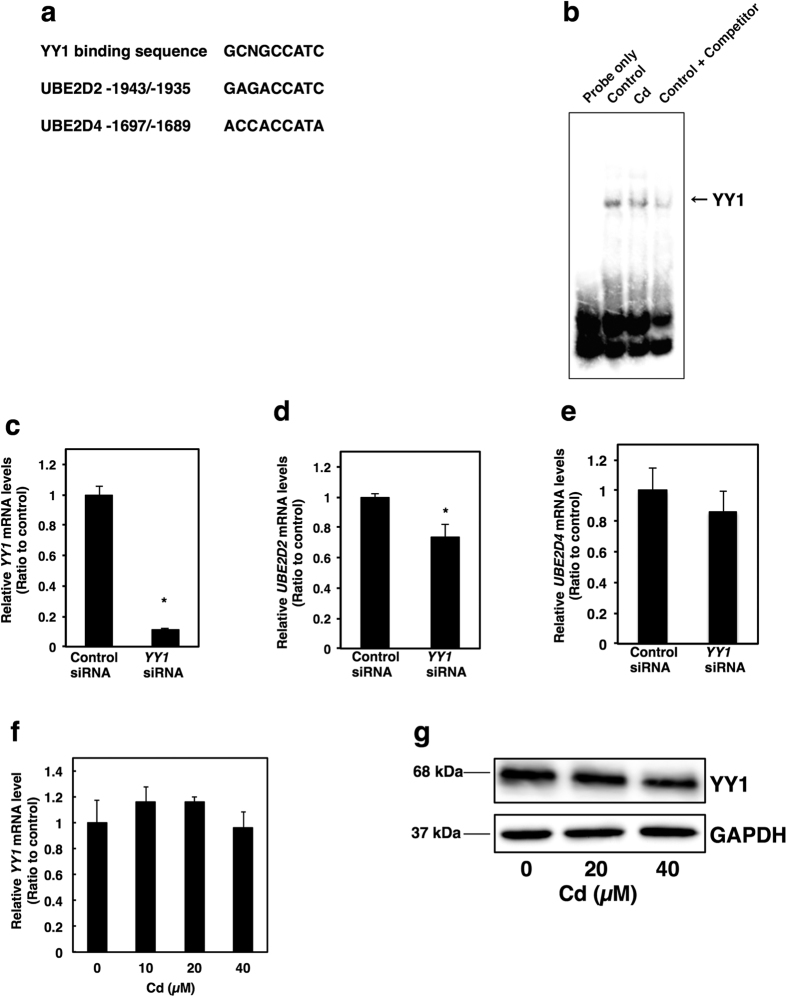
YY1 transcription factor is involved in Cd-induced suppression of *UBE2D2* gene expression in HK-2 cells. (**a**) High accuracy between the YY1 binding site and sequences upstream of *UBE2D2* and *UBE2D4* gene regions. Nucleotide abbreviation in the YY1 binding sequence is as follows: N is any nucleotide. (**b**) DNA binding activity of YY1 transcription factor in Cd-treated cells. (**c**) Knockdown efficiency of the *YY1* gene in HK-2 cells by *YY1* siRNA treatment. *YY1* siRNA was added to HK-2 cells for 24 h. (**d**,**e**) mRNA levels of *UBE2D2* and *UBE2D4* in HK-2 cells treated with *YY1* siRNA. *YY1* siRNA was added to HK-2 cells for 24 h. (**f**) Real-time RT-PCR of *YY1* gene expression. HK-2 cells were grown in six-well plates at a density of 2.0 × 10^4^ cells/cm^2^ and cultured for 48 h. Culture medium was discarded and the cells were treated with Cd (CdCl_2_) in serum-free culture medium for 3 h. (**c**–**f**) mRNA levels were examined using real-time RT-PCR. Values are the mean ± S.D. (*n* = 3). mRNA levels were normalized to *GAPDH*. *Significantly different from the control group, *P* < 0.05. (**g**) Western blot analysis of YY1 in HK-2 cells after treatment with Cd for 3 h. Anti-GAPDH antibody was used as a loading control. Upper panel, YY1; lower panel, GAPDH. The two blots were run under the same experimental conditions. Uncropped images are provided in Supplementary Fig. 1d.

**Figure 4:**
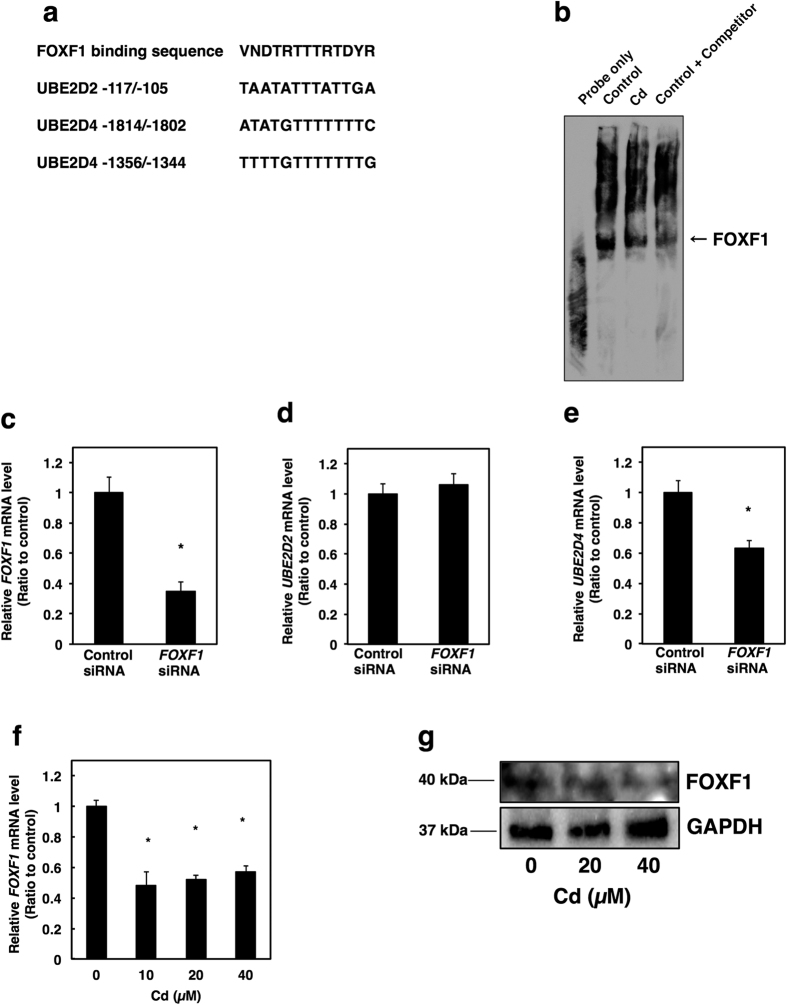
FOXF1 transcription factor is involved in Cd-induced suppression of *UBE2D4* gene expression in HK-2 cells. (**a**) High accuracy between the FOXF1 binding site and sequences upstream of *UBE2D2* and *UBE2D4* gene regions. Nucleotide abbreviation in the FOXF1 binding sequence is as follows: V, any nucleotide except T; N, any nucleotide; D, any nucleotide except C; R, A or G; and Y, C or T. (**b**) DNA binding activity of FOXF1 transcription factor in Cd-treated cells. (**c**) Knockdown efficiency of the *FOXF1* gene in HK-2 cells by *FOXF1* siRNA treatment. *FOXF1* siRNA was added to HK-2 cells for 24 h. (**d**,**e**) mRNA levels of *UBE2D2* and *UBE2D4* in HK-2 cells treated with *FOXF1* siRNA. *FOXF1* siRNA was added to HK-2 cells for 24 h. (**f**) Real-time RT-PCR of *FOXF1* gene expression. HK-2 cells were grown in six-well plates at a density of 2.0 × 10^4^ cells/cm^2^ and cultured for 48 h. Culture medium was discarded and the cells were treated with Cd (CdCl_2_) in serum-free culture medium for 3 h. (**c**–**f**) mRNA levels were examined using real-time RT-PCR. Values are the mean ± S.D. (*n* = 3). mRNA levels were normalized to *GAPDH*. *Significantly different from the control group, *P* < 0.05. (**g**) Western blot analysis of FOXF1 in HK-2 cells after treatment with Cd for 3 h. Anti-GAPDH antibody was used as a loading control. Upper panel, FOXF1; lower panel, GAPDH. The two blots were run under the same experimental conditions. Uncropped images are provided in Supplementary Fig. 1e.

It was investigated which transcription factors are involved in the Cd-induced suppression of *UBE2D2* and *UBE2D4* expression. Cd affected the transcriptional activity of transcription factors in NRK-52E cells in the previous study^39^. Using the protein/DNA binding assay, it was demonstrated that Cd increased the activities of 6 transcription factors by more than 2.0-fold and decreased those of 15 transcription factors by less than 0.5-fold among the 65 transcription factors monitored^39^. The core binding sequence of the transcription factor YY1 (Yin-Yang-1), the activity of which was most decreased by Cd treatment in NRK-52E cells, is GCNGCCATC (N is any nucleotide)^40^. High-accuracy sequences in the upstream of *UBE2D2* and *UBE2D4* gene regions were identified using the TRANSFAC^®^ database (BIOBASE GmbH) (Fig. 3a). Therefore, it was examined whether Cd affected the transcriptional activity of YY1 in HK-2 cells using the gel shift assay. Cd decreased the transcriptional activity of YY1 in HK-2 cells (Fig. 3b). Moreover, knockdown of *YY1* by siRNA transfection (Fig. 3c) decreased the gene expression of UBE2D2 (Fig. 3d), but not of UBE2D4 in HK-2 cells (Fig. 3e). Neither mRNA level nor protein level of YY1 transcription factor was affected by Cd treatment (Fig. 3f,g). These results suggest that Cd may suppress the gene expression of UBE2D2 *via* inhibition of YY1 transcription factor activity without the change of cellular level of YY1. In addition, we performed screening for transcription factors affected by Cd in HK-2 cells using the protein/DNA binding assay as previously described^39^. Among the transcription factors whose transcriptional activity decreased in the presence of Cd, transcription factor FOXF1 (forkhead box F1) was included (unpublished data). The core sequence for the binding of FOXF1 to DNA is VNDTRTTTRTDYR^41^. The core sequences were found in the upstream of *UBE2D2* and *UBE2D4* gene regions, using the TRNASFAC^®^ database (Fig. 4a). Next, we examined the effect of Cd on the transcriptional activity of FOXF1 and the effect of knockdown of *FOXF1* on the gene expression of UBE2D4. Cd suppressed the transcriptional activity of FOXF1 by using the gel shift assay (Fig. 4b). Furthermore, gene expression of UBE2D4, but not that of UBE2D2, was inhibited by siRNA transfection against the *FOXF1* gene (Fig. 4c–e). Cd decreased both mRNA level and protein level of FOXF1 (Fig. 4f,g). These results suggest that Cd may suppress the gene expression of UBE2D4 *via* inhibition of FOXF1 transcription factor activity through the decrease in cellular level of FOXF1.

### Cd induces apoptosis through overaccumulation of p53 protein in HK-2 cells

**Figure 5:**
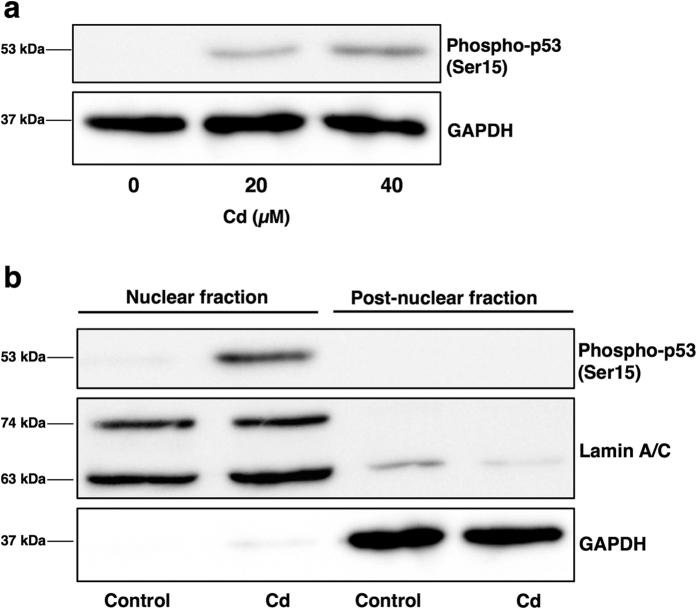
Cd increases phosphorylated p53 protein levels in HK-2 cells. (**a**) Phosphorylated p53 protein levels in Cd-treated HK-2 cells. Whole cell lysates of HK-2 cells treated with 20 or 40 μM Cd (CdCl_2_) for 6 h were used for western blot analysis and probed with phospho-p53 antibody. Anti-GAPDH antibody was used as a loading control. Upper panel, phosphor-p53; lower panel, GAPDH. The two blots were run under the same experimental conditions. Uncropped images are provided in Supplementary Fig. 1f. (**b**) Localization of phosphorylated p53 protein in Cd-treated HK-2 cells. HK-2 cells were treated with 40 μM Cd for 3 h and separated into nuclear and post-nuclear fractions. Western blotting for the detection of phospho-p53 was performed (upper panel). Lamin A/C, nuclear marker (middle panel); GAPDH, cytosolic marker (lower panel). The three blots were run under the same experimental conditions. Uncropped images are provided in Supplementary Fig. 1g.

**Figure 6:**
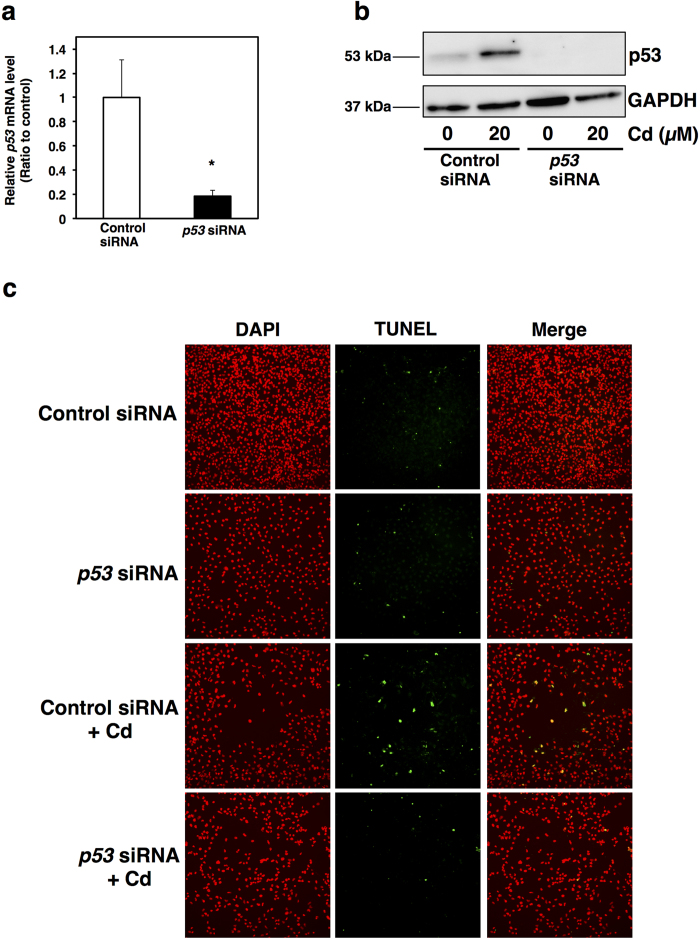
Knockdown of *p53* decreases Cd-induced apoptosis in HK-2 cells. (**a**) Knockdown efficiency of *p53* in HK-2 cells following *p53* siRNA treatment. *p53* siRNA was added to HK-2 cells and incubated for 24 h. mRNA levels were examined using real-time RT-PCR. mRNA levels were normalized to *GAPDH*. Values are the mean ± S.D. (*n* = 3). *Significantly different from the control group, *P* < 0.05. (**b**) Protein levels of p53 in HK-2 cells treated with *p53* siRNA and Cd (CdCl_2_). HK-2 cells were treated with or without *p53* siRNA for 24 h, and then treated with or without 20 μM Cd for 18 h. Whole cell lysates of HK-2 cells were used for western blot analysis. Anti-GAPDH antibody was used as a loading control. Upper panel, p53; lower panel, GAPDH. The two blots were run under the same experimental conditions. Uncropped images are provided in Supplementary Fig. 1h. (**c**) Representative images of TUNEL-positive apoptotic cell (green signals). HK-2 cells were treated with or without *p53* siRNA for 24 h, and then treated with or without 20 μM Cd for 18 h. Nuclei were counterstained with DAPI, and the blue signal was converted to red (DAPI). Fluorescein signals from TUNEL-positive cells (TUNEL) and red signals of nuclei were merged (Merge). Original magnification: × 100.

**Figure 7:**
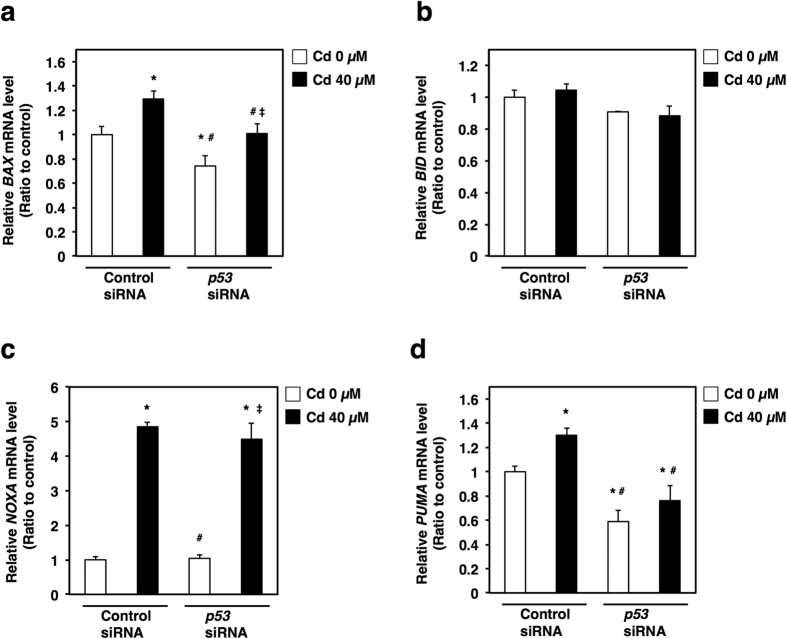
Cd-induced accumulation of p53 protein induces apoptosis *via* increases in apoptosis-related gene expression in HK-2 cells. Real-time RT-PCR of *BAX* (**a**), *BID* (**b**), *NOXA* (**c**) and *PUMA* (**d**) gene expression. HK-2 cells were treated with or without *p53* siRNA for 24 h, and then treated with or without 40 μM Cd (CdCl_2_) for 6 h. mRNA levels were examined using real-time RT-PCR. Values are the mean ± S.D. (*n* = 3). mRNA levels were normalized to *GAPDH*. Values are the mean ± S.D. (*n* = 3). *Significantly different from “the treatment group of control siRNA and 0 μM Cd”, *P* < 0.05. ^#^Significantly different from “the treatment group of control siRNA and 40 μM Cd”, *P* < 0.05. ^‡^Significantly different from “the treatment group of *p53* siRNA and 0 μM Cd”, *P* < 0.05.

The phosphorylated form of nuclear p53 is very important for nuclear retention and subsequent transcriptional activity for genes involved in cell cycle arrest and apoptosis^42^. Cd-increased phosphorylated p53 is present not only in whole cell lysates, but also in the nuclear fraction (Fig. 5a,b). It is considered that Cd-induced accumulation and phosphorylation of p53 promote p53 transcriptional activity in HK-2 cells. To define the actual role of p53 accumulation in Cd-induced apoptosis, we next monitored apoptosis using TUNEL staining in Cd-treated *p53*-knockdown cells. Transfection of siRNA against *p53* significantly decreased the transcription and cellular protein levels of p53 (Fig. 6a,b). As shown in Fig. 6c, Cd increased TUNEL-positive staining in control siRNA transfected cells. However, knockdown of *p53* decreased the number of cells undergoing apoptosis following Cd treatment (Fig. 6c). p53 protein is a transcription factor that regulates the transcription of apoptosis-related genes such as *BAX*, *BID*, *NOXA* and *PUMA*^43^,^44^. Therefore, it was examined which genes were related to Cd-induced apoptosis through the overaccumulation of p53 in HK-2 cells. Cd significantly increased the gene expression of BAX (BCL2-associated X protein), NOXA and PUMA, but not BID (BH3 interacting domain death agonist) (Fig. 7). Knockdown of *p53* by siRNA decreased the basal levels of *BAX* and *PUMA* gene expression (Fig. 7a,d). Although Cd increased the gene expression of BAX in *p53*-knockdown cells, the expression levels of *BAX* when treated with Cd and *p53* siRNA were lower than that of *BAX* when treated with Cd and control siRNA (Fig. 7a). However, the gene expression level of PUMA was not increased by Cd treatment in *p53*-knockdown cells (Fig. 7d). Knockdown of *p53* did not affect gene expression levels of NOXA with or without Cd (Fig. 7c). These findings indicate that elevation of intracellular p53 protein levels is associated with Cd-induced apoptosis *via* its transcriptional activity in HK-2 cells.

### Cd induces both p53 accumulation and apoptosis in proximal tubular cells *in vivo*

**Figure 8:**
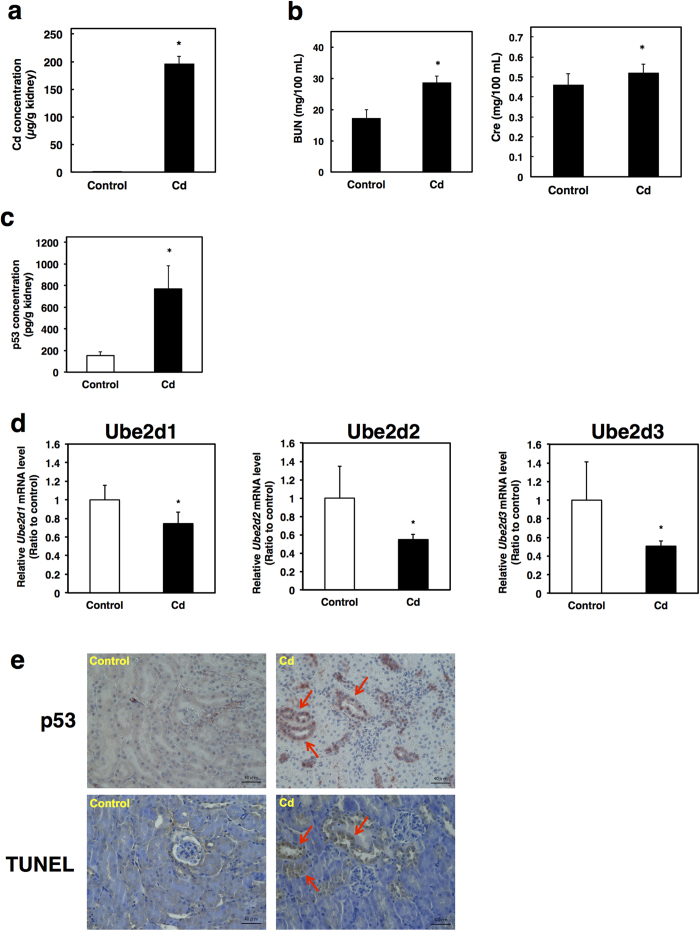
Cd induces both p53 accumulation and apoptosis in renal proximal tubular cells *in vivo*. (**a**) Cd accumulation in the kidneys of mice exposed to Cd for 6 months. A portion of the kidney was obtained after 6-month Cd exposure. The kidney was digested with nitric acid hydrogen peroxide and the Cd content was measured by inductively coupled plasma mass spectrometry (ICP-MS). Cd content was normalized to the total amount of protein. (**b**) Changes in parameters of renal toxicity. Blood urea nitrogen (BUN) and creatinine levels (Cre) in serum were measured using the automatic dry-chemistry analyzer system. (**c**) Protein levels of p53 in the kidney mice exposed to Cd for 6 months. Protein levels of p53 were determined using the ELISA assay. (**d**) UBE2D family gene expression in the kidneys of mice exposed to Cd for 6 months. mRNA levels were measured by real time RT-PCR and normalized to *ß-actin*. (**a**–**d**) Values are the mean ± S.D. (*n* = 5 or 6). *Significantly different from the corresponding control group, *P* < 0.05. (**e**) Representative images of p53 staining and TUNEL staining. Immunohistochemical detection of p53 was performed using the anti-p53 antibody (upper panels) and detection of apoptosis was conducted by colorimetric detection of TUNEL-positive signals (lower panels). Serial sections of the mouse kidney were obtained from mice exposed to Cd for 6 months. Serial sections of kidney tissue from control mice are shown in the left panel and those of Cd-exposed mice in the right panel. Red arrows indicate p53 expression (upper panel) and apoptotic cells (lower panel). Original magnification: × 400.

Long-term exposure to Cd is known to damage renal proximal tubule function^1^,^2^,^3^,^4^,^5^,^6^. Therefore, we examined whether Cd-induced apoptosis occurred following accumulation of p53 in the kidney of mice. Mice were exposed to 300 ppm Cd *via* chow for 6 months. After treatment, the Cd concentration in the kidney was 196.60 ± 13.15 μg/g tissue (Fig. 8a). In the kidney of mice exposed to Cd (300 ppm) for 3 months, the Cd concentration was 97.56 ± 9.48 μg/g tissue (unpublished data). Long-term exposure (6 months) to 300 ppm Cd *via* chow has been shown to accumulate to critical levels in the kidney of mice, as the ratio of gastrointestinal absorption of Cd is 1–2% in mice and rats^45^. Cd slightly elevated blood urea nitrogen (BUN) levels and serum creatinine levels (Fig. 8b). Because the critical concentration of Cd in the mouse kidney is known to be 200 μg/g tissue^1^,^46^, Cd (300 ppm) exposure for 6 months is thought to induce kidney toxicity. ELISA analysis showed that p53 protein was markedly increased in the kidney of Cd-exposed mice compared with control mice (Fig. 8c). In the kidney of mice exposed to Cd, the expression of UBE2D family genes was significantly decreased compared with control mice (Fig. 8d). These results indicate that Cd-exposure for 6 months *via* chow not only increases p53 protein levels in the kidney, but also decreases the expressions of UBE2D family genes as shown in HK-2 cells. Immunostaining analysis of the kidney sections with anti-p53 antibody indicated that proximal tubular cells were predominantly p53-positive in Cd-exposed mice compared with control mice (Fig. 8e). Interestingly, both TUNEL-positive staining and p53-positive staining were detected in identical proximal tubular cells of the kidney of mice exposed to Cd (Fig. 8e). However, neither p53-stained cells nor TUNEL-positive cells were detected in the glomeruli of both Cd-exposed and control mice (Fig. 8e). These findings clearly indicate that Cd excessively accumulates in the kidney, especially in proximal tubules, and induces apoptosis mediated by the accumulation of p53 protein.

## Discussion

Cd is a toxic chemical that adversely affects kidney function. Apoptosis is thought to be a cell death program that Cd induces in various cell types^4^. Our present study reveals that Cd induces apoptosis, and that p53 is involved in Cd-induced apoptosis in HK-2 cells. The tumor suppressor p53 is involved in the inhibition of cell growth and apoptosis through transcriptional activity^31^. Although several studies suggest the involvement of p53 in Cd toxicity in various cells^26^,^27^,^28^,^29^,^30^, the previous reports revealed that only the protein levels of p53 or phosphorylated p53 were affected by Cd treatment^26^,^27^, and other studies used different cells from proximal tubular cells those are main target of Cd toxicity^29^,^30^. Differently from previous reports, the present study shows that p53 is directly involved in Cd-induced apoptosis *via* transcriptional activity in HK-2 cells by using siRNA transfection. Moreover, apoptosis and accumulation of p53 were detected in the same spots of renal proximal tubules in Cd-exposed mice. These results strongly indicate that p53 is a critical factor in the pathway of Cd-induced apoptosis in the kidney.

In the present study, we have for the first time identified that the down-regulation of *UBE2D2* and *UBE2D4* genes is critical for Cd-induced accumulation of intracellular p53 protein in human proximal tubular cells. In rat proximal tubular cells and mouse kidney, Cd decreased the gene expression of the UBE2D family and resulted in p53 protein accumulation^16^. However, the previous study did not offer direct evidence linking the regulation of p53 levels upon Cd-treatment with the UBE2D family.

YY1 and FOXF1 were identified as the transcription factors involved in the suppression of *UBE2D2* and *UBE2D4* gene expression by Cd in HK-2 cells (Figs 3 and 4). The putative sequences for binding of YY1 and FOXF1 exist upstream of both *UBE2D2* and *UBE2D4*. However, YY1 affected *UBE2D2* gene expression and FOXF1 affected *UBE2D4* gene expression. These results suggest that Cd may affect gene expressions through the activities of specific transcription factors through various pathways. Moreover, in this study, the effects of Cd on the mRNA levels and protein levels of YY1 and FOXF1 were determined. Cd decreased the cellular level of FOXF1, but not that of YY1 (Figs 3 and 4). YY1 was found as the substrate for Plk1 (Polo-like kinase 1) and Aurora B kinase^47^,^48^. Plk1 and Aurora B phosphorylate YY1 at G2/M stage of the cell cycle^47^,^48^. Cd was reported to arrest G2/M stage dependent on kinase signaling in NRK-52E cells^49^. Previous report suggested that nucleo-cytoplasmic shuttling of FOXF1 is dynamically regulated^50^. Cd also affected intracellular localization of proteins in NRK-52E cells^51^,^52^. In addition, YY1 and FOXF1 need to interact with other factors to bind to DNA^53^,^54^,^55^. Therefore, Cd may regulate the transcriptional activities of YY1 and FOXF1 *via* not only cellular protein level but also unknown effects on their transcription abilities, thereby inhibiting *UBE2D2* and *UBE2D4* expression. The precise mechanism of Cd-regulated activities of transcription factors will be an interesting issue and needs further studies to address it.

In unstressed cells, ubiquitination of p53 and degradation primarily occur in the cytoplasm^56^,^57^. Although undegraded p53 is imported into the nucleus, MDM2 located in the nucleus mediates the export of p53 to the cytoplasm and cytoplasmic degradation. After stress, non-ubiquitinated p53 accumulates in the cytoplasm, and phosphorylation of p53 inhibits the binding of MDM2 to p53^42^,^57^. Moreover, Cd markedly increased intranuclear phosphorylated-p53 levels (Fig. 5b). These results strongly indicate that Cd not only inhibits the expression of UBE2D family genes but also promotes the activity of kinases. Cd is also reported to activate the MAPK cascade, one of the pathways of p53 phosphorylation^58^,^59^.

Phosphorylation of p53 is considered a crucial step in p53 stabilization^42^,^57^,^60^. N-terminal phosphorylation at Ser15 is thought to stabilize p53 by inhibiting the interaction between p53 and MDM2^42^,^60^. Stabilized p53 in the nucleus confers the activity of the transcription factor. Target genes of p53 encode apoptotic proteins such as BAX, BID, NOXA, and PUMA^43^,^44^. In the present study, phosphorylated p53 completely localized to the nuclear fraction following Cd treatment (Fig. 5b). Moreover, our study suggests that BAX and PUMA are the p53 response elements in Cd-induced apoptosis (Fig. 7). The target genes of p53 depend on its modification^61^. The modification of Lys164 residues within p53 induces *PUMA* expression, while modification of Lys372 induces *BAX* expression^61^. Cd may trigger spontaneous modification of p53, as well as phosphorylation. Taken together, our study indicates that Cd-induced apoptosis is dependent on the transcriptional activity of stabilized p53 in the nucleus of HK-2 cells.

Cd decreased *UBE2D2* and *UBE2D4* mRNA levels, but not *UBE2D1* and *UBE2D3* mRNA levels in HK-2 cells. A previous study demonstrated that UBE2D2 and UBE2D3 regulated cellular p53 protein levels in MCF7 cells^32^. However, the present study showed that *UBE2D2* and *UBE2D4* mainly relate to Cd-induced accumulation of p53 in HK-2 cells. In addition, inorganic arsenic increased intracellular p53 protein levels through the suppression of *UBE2D2* and *UBE2D4* genes^22^. Moreover, some researchers have reported that the inhibition or induction of a specific gene or protein is dependent upon the chemical substance, tissue and cell type^62^,^63^. Therefore, Cd responsive genes may vary in different species, and different pathways may be involved in the regulation of p53-dependent apoptotic cell death in different cell types as well as different stressors.

Our present study demonstrated that down-regulation of UBE2D family genes followed by accumulation of p53 in proximal tubular cells is an important mechanism for Cd-induced renal toxicity. In addition, YY1 and FOXF1 were identified as the target transcription factors involved in Cd-induced down-regulation of UBE2D family genes. In this study, the precise mechanism of Cd toxicity *via* the suppression of UBE2D family gene expression may be illustrated by the studies from the transcription factors to p53-related apoptosis.

## Materials and Methods

### Cell culture

HK-2 cells were kindly provided by Dr. Takada (Jikei University School of Medicine, Tokyo, Japan). HK-2 cells were cultured in Dulbecco’s Modified Eagle’s Medium/Ham’s Nutrient Mixture F-12 (Sigma-Aldrich, St. Louis, MO, USA) supplemented with 10% (v/v) fetal bovine serum (Gibco, Grand Island, NY, USA), 25 U/mL penicillin (DS Pharm, Osaka, Japan), 25 μg/mL streptomycin (DS Pharm), 1% (v/v) Insulin-Transferrin-Selenium-X (Gibco), 10 ng/mL epidermal growth factor (EGF; Sigma-Aldrich) and 5 ng/mL hydrocortisone (Sigma-Aldrich) at 37 °C in a humidified incubator containing 5% (v/v) CO_2_.

### Cell viability

HK-2 cells were grown in 96-well plates at a density of 2.0 × 10^4^ cells/cm^2^ and cultured until confluent. The culture medium was discarded and the cells were treated with Cd (CdCl_2_) (Wako Pure Chemical Industries, Osaka, Japan) in serum-free culture medium for 6 or 24 h. After treatment, the serum-free medium containing Cd was replaced with fresh growth medium containing 10% (w/v) Alamar Blue (Invitrogen, Grand Island, NY, USA) and incubated for 4 h at 37 °C. Fluorescence was measured at excitation wavelength of 540 nm and an emission wavelength of 595 nm (DTX880 multimode detector; Beckman Coulter Inc., Brea, CA, USA).

### Western blot analysis

After treatment, cells were washed twice with ice-cold phosphate-buffered saline (PBS) and lysed in RIPA buffer [25 mM Tris (pH7.6), 150 mM NaCl, 1% NP-40, 1% sodium deoxycholate 0.1% SDS; Thermo Fisher Scientific, Waltham, MA, USA]. The protein concentration was measured using the BCA protein assay kit (Thermo Fisher Scientific). Protein was electrophoresed on SDS-polyacrylamide gels and transferred to a polyvinylidene difluoride membrane. The membrane was probed with antibodies against p53 (1:1000; Cell Signaling Technology, Danvers, MA, USA), phospho-p53 (Ser15) (1:1000; Cell Signaling Technology), MDM2 (1:200; Santa Cruz Biotechnology, Dallas, TX, USA), YY1 (1:200; Santa Cruz Biotechnology), FOXF1 (1:500; Santa Cruz Biotechnology), GAPDH (1:2000; American Research Products, Waltham, MA, USA) or Lamin A/C (1:1000; Cell Signaling Technology). The membrane was subsequently probed with horseradish peroxidase-conjugating (HRP) secondary anti-rabbit antibody, anti-mouse antibody (1:10000; GE Healthcare, Tokyo, Japan), or anti-goat antibody (1:10000; Dako Denmark A/S, Glostrup, Denmark); and protein was detected by enhanced chemiluminescence using Chemi-Lumi One L (Nacalai Tesque, Kyoto, Japan). The chemiluminescence images were taken using the LAS-3000 (Fujifilm, Tokyo, Japan) device.

### Real time RT-PCR

Total RNA of HK-2 cells or mouse tissue was extracted with the Quick Gene RNA cultured cell kit S or RNA tissue kit S (Fujifilm), respectively. Total RNA was incubated with the PrimeScript reverse transcription (RT) Reagent Kit (Perfect Real Time) (Takara Bio, Shiga, Japan) to generate cDNA. Real-time PCR was performed with SYBR Premix Ex TaqII (Perfect Real Time) (Takara Bio) and the Thermal Cycler Dice Real time System (Takara Bio). PCR conditions were as follows: 10 s hot-start at 95 °C followed by 40 cycles of 5 s at 95 °C and 30 s at 60 °C. Gene expression was normalized to *GAPDH* or *ß-actin* mRNA levels. The oligonucleotide sequences of the primers were as follows: sense, 5′-GAGTAATTTGGGGTTTGTCTTGG-3′, and antisense, 5′-CCTTTCTTTTGGATGGGTGAT-3′, for the human *UBE2D1* gene; sense, 5′-TTGTCCATCTGTTCTCTGTTGTG-3′,and antisense, 5′-TCCATTCCCGAGCTATTCTGT-3′, for the human *UBE2D2* gene; sense, 5′-GGTGCAGCCCCTGTCTAACT-3′, and antisense, 5′-GGCCTTGTAGGTGTGTGCTATCTC-3′, for the human *UBE2D3* gene; sense, 5′-TGGTCTCCAGCGTTGACTG-3′, and antisense, 5′-GGCCTTGTAGGTGTGTGCTATCTC-3′, for the human *UBE2D4* gene; sense, 5′-CCTATCCGGTCAGTTGTTGGA-3′, and antisense, 5′-TTGCAGAGTGGAGGAAATGG-3′, for the human *p53* gene; sense, 5′-GAAGGAAACTGGGGAGTCTTG-3′, and antisense, 5′-GCTGGAATCTGTGAGGTGGT-3′, for the human *MDM2* gene; sense, 5′-ATTGCTGTGCAGAACCCTCT-3′, and antisense, 5′-TGCAACTCCTTGCTGTCATC-3′, for the human *OTUB1* gene; sense, 5′-GAGGAGGACATGGAGGATGA-3′, and antisense, 5′-TTTGGTGTGGTCTGTCTGGA-3′, for the human *USP7* gene; sense, 5′-GGGGACGAACTGGACAGTAA-3′, and antisense, 5′-CAGTTGAAGTTGCCGTCAGA-3′, for human *BAX* gene; 5′-AAGAAGGTGGCCAGTCACAC-3′, and antisense, 5′-GTCCATCCCATTTCTGGCTA-3′, for the human *BID* gene; sense, 5′-AGCTGGAAGTCGAGTGTGCT-3′, and antisense, 5′-TCCTGAGCAGAAGAGTTTGGA-3′, for the human *NOXA* gene; sense, 5′-GACGACCTCAACGCACAGTA-3′, and antisense, 5′-CTGGGTAAGGGCAGGAGTC-3′, for the human *PUMA* gene; sense, 5′-AAACATCTGCACACCCACGG-3′, and antisense, 5′-TTCCCACAGCCTTCGAACGT-3′, for the human *YY1* gene; sense, 5′-ATTCCTACATCGCGCTCATC-3′, and antisense, 5′-TGATGAAGCACTCGTTGAGC-3′, for the human *FOXF1* gene; sense, 5′- GCACCGTCAAGGCTGAGAAC-3′, and antisense, 5′-TGGTGAAGACGCCAGTGGA-3′, for the human *GAPDH* gene; sense, 5′-ACAACAGGCACGCAAGAG-3′, and antisense, 5′-GAACGAAGGACACGGCAAAC-3′, for the mouse *Ube2d1* gene; sense, 5′-ATGCAAAGGTGTTGGTTGCT-3′, and antisense, 5′-TGCTCATGTTTCCCAGGTTC-3′, for the mouse *Ube2d2* gene; sense, 5′-CAGTAATGGCAGCATTTGTCTTG-3′, and antisense, 5′-GGGTCGTCTGGGTTTGGAT-3′, for the mouse *Ube2d3* gene; sense, 5′-CCTAAGGCCAACCGTGAAAA-3′, and antisense, 5′-AGCCATACAGGGACAGCACA-3′, for the mouse *ß-actin* gene.

### siRNA transfection

Silencer Select Pre-designed siRNAs were purchased from Ambion (Grand Island, NY, USA). The I.D. of siRNAs were as follows: s605 (Silencer^®^ Select Validated siRNA), for human *p53*; s14575 (Silencer^®^ Select Validated siRNA), for human *UBE2D2*; s224224 (Silencer^®^ Select Pre-designed siRNA), for human *UBE2D4*; 11538, n337458 and s14960 (Silencer^®^ Select Pre-designed siRNA), for human *YY1*; s5220, s5221 and s5222 (Silencer^®^ Select Pre-designed siRNA), for human *FOXF1*. siRNA transfection was performed using Lipofectamine RNAiMAX (Invitrogen). After the siRNA mixture was incubated for 15 min with Lipofectamine RNAiMAX and Opti-MEM^®^ I Reduced Serum Medium (Opti-MEM; Gibco), HK-2 cells were transfected with the siRNA mixture for 24 h.

### Detection of apoptosis

HK-2 cells were transferred to Millicell EZ SLIDE (Millipore, Billerica, MA, USA) at a density of 2.0 × 10^4^ cells/cm^2^ for 24 h and then the siRNA mixture [1 nM siRNA/sequence, 0.2% (v/v) Lipofectamine RNAiMAX, 10% (v/v) Opti-MEM] was added. After 24 h, the cells were incubated with or without 20 μM Cd in serum-free culture medium for 18 h. Apoptotic cells were detected using the *In Situ* Cell Death Detection Kit, Fluorescein (Roche, Mannheim, Germany) according to the manufacturer’s protocol. Fluorescence images were taken using a BIOREVO BZ-9000 microscope (Keyence, Osaka, Japan).

### Nuclear extraction

Nuclei were extracted using the Nuclear Extraction Kit (Panomics; Affymetrix, Santa Clara, CA, USA). HK-2 cells were treated with 40 μM Cd for 3 h. After the treatment, the cells were washed twice with ice-cold PBS, harvested from the plates and lysed on ice for 10 min in extraction kit buffer A containing protease inhibitors, phosphatase inhibitors, and dithiothreitol (DTT) with gentle shaking. Nuclei were collected by centrifugation at 14,000 × g for 3 min at 4 °C. The pellet was resuspended in extraction buffer B containing protease inhibitors, phosphatase inhibitor and DTT, and incubated at 4 °C with gentle shaking. The suspension was then centrifuged at 14,000 × g for 5 min at 4 °C, and the supernatant was collected as the nuclear extraction.

### Gel shift assay

The gel shift assay was performed using the EMSA kit purchased from Panomics. Nuclear extractions from control and Cd-treated HK-2 cells were used for the assay. The protein concentration of nuclei was measured using the BCA protein assay kit. Nuclear protein (3 μg) was incubated with 10 ng DNA probe (biotin-labeled binding sequence to transcription factor) and 1 μg poly d(I-C) with binding buffer for 30 min at 15 °C in a thermal cycler (Takara Bio). For the competition assay, 1,320 ng cold DNA probe was added. The protein-bound probe was electrophoresed on a 5.0% (w/v) TBE [Tris borate-ethylenediaminetetraacetic acid (EDTA)]-polyacrylamide gel in 0.5 × TBE buffer at 4 °C and then transferred to a Biodyne^®^ B nylon membrane (Pall corporation, Port Washington, NY, USA) in 0.5 × TBE buffer. The membrane was fixed by UV crosslinking (CL-1000 Ultraviolet Crosslinker; UVP, Upland, CA, USA) with 120 mJ/cm^2^. The membrane was blocked and probed with Streptavidin-HRP. The chemiluminescence images were taken using a LAS-3000 device.

### Animals

All animal experiments were performed in accordance with the Regulation on Animal Experimentation at School of Pharmacy, Aichi Gakuin University, Nagoya, Aichi, Japan. All procedures to maintain and use mice were approved by the Animal Care and Use Committee for School of Pharmacy, Aichi Gakuin University, Nagoya, Aichi, Japan. Four-week-old female C57BL/6J mice were purchased from CLEA Japan (Tokyo, Japan) and routinely bled in the vivarium of the laboratory animal facility of Aichi Gakuin University. The mice were housed in cages in a ventilated animal room at 23 ± 1 °C with relative humidity, and a 12 h light–dark cycle. After the adaptation period, five-week-old mice were randomly assigned to control and experimental groups, with 5–6 mice per group. Control and Cd-exposed groups were fed standard laboratory chow or 300 ppm Cd-containing chow (Oriental-BioService, Kyoto, Japan), respectively, and had access to tap water *ad libitum*. After 6 months of Cd-exposure, the blood and kidneys were collected from each mouse under ether anesthesia.

### p53 protein level

The kidney was homogenized in nine volumes of low-salt RIPA buffer [20 mM Tris, 0.5 mM EDTA, 1% NP40, 0.5% sodium deoxycholate, 0.05% SDS, 1 mM phenylmethylsulfonyl fluoride (PMSF), 1μg/mL aprotinin, 2 μg/mL leupeptin] and the homogenate was centrifuged at 10,000 × *g* for 10 min at 4 °C. Protein levels of p53 in the supernatant were quantified using a p53 Enzyme Immunometric Assay Kit (Assay Designs, Ann Arbor, MI, USA). Total protein concentration was measured to normalize p53 levels.

### Determination of Cd content

A portion of the kidney was digested with nitric acid and hydrogen peroxide. The inorganic residues and internal standard (20 ppb yttrium solution) were dissolved in ultrapure water, and Cd and yttrium content were measured by inductively coupled plasma mass spectrometry (ICP-MS; Agilent Technologies, Santa Clara, CA, USA). Cd content was adjusted to yttrium content.

### Evaluation of renal toxicity

Blood urea nitrogen (BUN) and creatinine values in the serum were measured as indicators of renal toxicity using the automatic dry-chemistry analyzer system (Spotchem EZ SP-4430; Arkray, Kyoto, Japan).

### Detection of apoptosis and p53 in tissue sections

The kidney was fixed in 10% (v/v) neutral buffered formalin solution and embedded in paraffin. Deparaffinized serial tissue sections of 5 μm thickness were used for immunohistochemical staining of p53 and apoptosis detection. For immunohistochemical staining of p53, antigen retrieval was conducted for 30 min at 90 °C in 0.1 M boric acid pH 7.0. After antigen retrieval, kidney sections were quenched in 0.3% (v/v) hydrogen peroxide for 10 min and them stained using anti-p53 antibody (Abcam, Cambridge, MA, USA). Immunohistochemical detection of p53 antibody was performed using HistoMouse™-*Plus* Kit (Invitrogen) according to the manufacturer’s protocol. Apoptosis was detected using the TACS^®^ 2 TdT-DAB *In situ* Apoptosis Detection Kit (Trevigen, Gaithersburg, MD, USA). Briefly, deparaffinized sections were digested with Cytonin™ for 30 min, and rinsed with dH_2_O twice, 2 min each. The sections were quenched in 0.3% (v/v) hydrogen peroxide for 5 min, followed by incubation in TdT (terminal deoxynucleotidyl transferase) enzyme solution for 1 h at 37 °C in a humidity chamber. The labeling reaction was stopped using TdT Stop Buffer for 5 min at room temperature, and rinsed with dH_2_O twice, 5 min each. Streptavidin-HRP solution was applied to the sections for 10 min at 37 °C, and rinsed with PBS twice, 2 min each. The positive signal was developed using diaminobenzidine solution for 7 min. After washing with dH_2_O several times, the sections were counterstained with 1% (v/v) methyl green and mounted with coverslips. Microscopy images were obtained using a BIOREVO BZ-9000 microscope.

### Statistical analysis

Statistical analyses were performed using one-way analysis of variance (ANOVA). When the F value was significant (*P* < 0.05), Bonferroni’s multiple *t*-test was performed for post-hoc comparison (*P* < 0.05). Student *t*-test was performed for two-sample comparison (*P* < 0.05).

## Additional Information

**How to cite this article**: Lee, J.-Y. *et al.* Accumulation of p53 via down-regulation of UBE2D family genes is a critical pathway for cadmium-induced renal toxicity. *Sci. Rep.* **6**, 21968; doi: 10.1038/srep21968 (2016).

## Supplementary Material

Supplementary Information
