# Supplementary material for: Accumulation of p53 via down-regulation of UBE2D family genes is a critical pathway for cadmium-induced renal toxicity
Source: Sci Rep. 2016 Feb 25;6:21968. doi: 10.1038/srep21968 (PMC4766413; doi:10.1038/srep21968)

Supplementary Information of:

Accumulation of p53 via down-regulation of UBE2D family genes is a critical pathway for cadmium-induced renal toxicity

Jin-Yong Lee[1], Maki Tokumoto[1], Yasuyuki Fujiwara[1,2], Tatsuya Hasegawa[3], Yoshiyuki Seko[3], Akinori Shimada[4] and Masahiko Satoh[1]*

[1] Laboratory of Pharmaceutical Health Sciences, School of Pharmacy, Aichi Gakuin University, 1-100 Kusumoto-cho, Chikusa-ku, Nagoya, Aichi 464-8650, Japan

[2] Department of Environmental Health, School of Pharmacy, Tokyo University of Pharmacy and Life Sciences, 1432-1 Horinouchi, Hachioji, Tokyo 192-0392, Japan

[3] Department of Environmental Biochemistry, Mount Fuji Research Institute, 5597-1 Kenmarubi, Kamiyoshida, Fujiyoshida, Yamanashi 403-0005, Japan

[4] Laboratory of Pathology, Department of Medical Technology, School of Life and Environmental Science, Azabu University, 1-17-71 Fuchinobe, Chuo-ku, Sagamihara, Kanagawa 252-5201, Japan

---

*Address for all correspondence:
Masahiko Satoh, Ph.D.
Laboratory of Pharmaceutical Health Sciences, School of Pharmacy, Aichi Gakuin University, 1-100 Kusumoto-cho, Chikusa-ku, Nagoya, Aichi 464-8650, Japan
E-mail: masahiko@dpc.agu.ac.jp

Supplementary Figure 1. Original images of cropped blots.

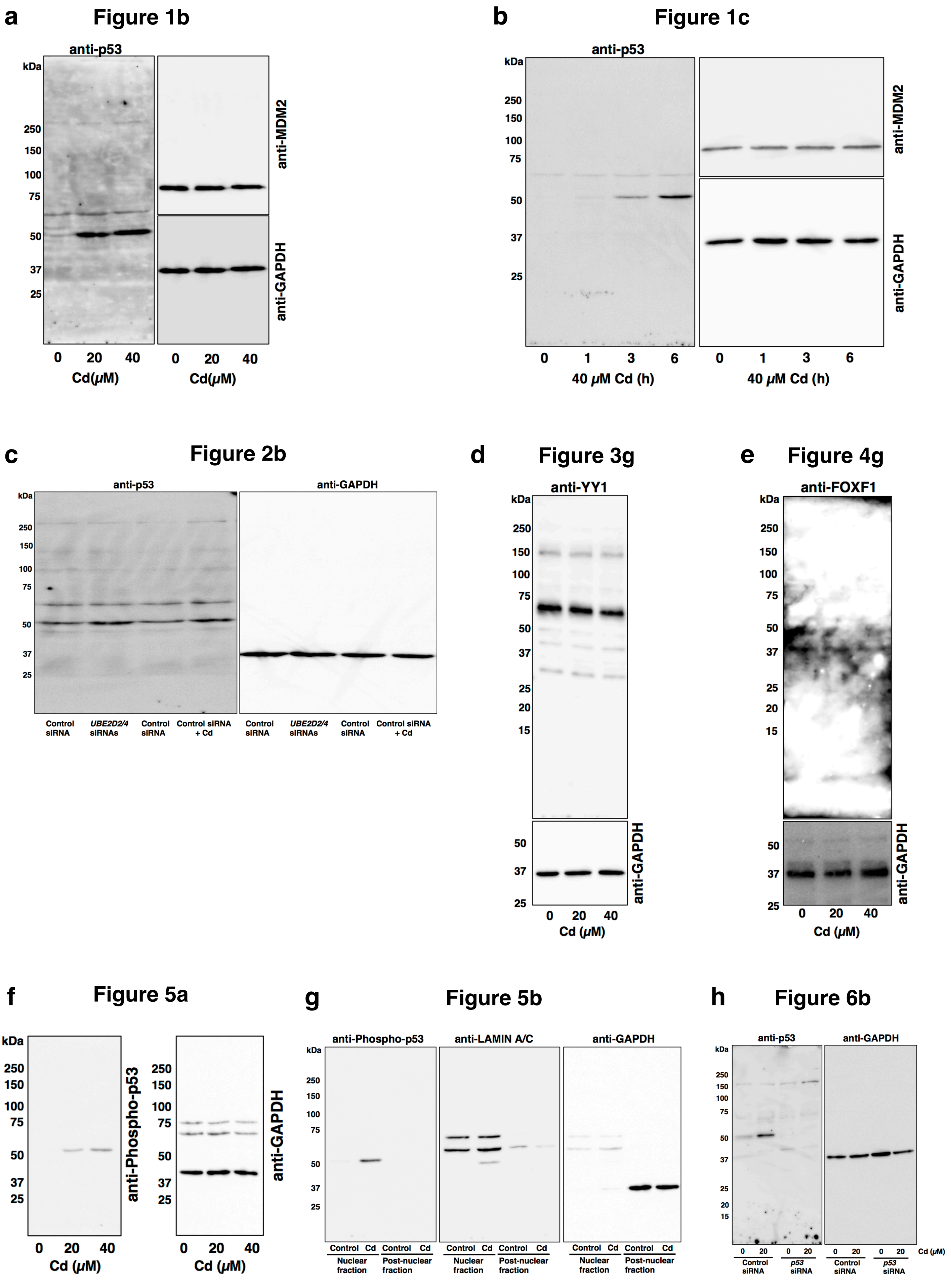

Supplement: Supplementary Information [file srep21968-s1.pdf]
